# Pulmonary thromboembolism in children with rheumatic diseases

**DOI:** 10.1186/1546-0096-10-S1-A73

**Published:** 2012-07-13

**Authors:** Larry B Vogler, Sheila Angeles-Han, Sampath Prahalad, Egla C Rabinovich

**Affiliations:** 1 Duke University Medical Center, Durham, NC, USA; 2 Emory Children's Center, Atlanta, GA, USA; 3 Emory University, Atlanta, GA, USA; 4 Emory University School of Medicine, Atlanta, GA, USA

## Purpose

To demonstrate the clinical features and predisposing factors of pulmonary thrombotic events in children with rheumatic diseases.

## Methods

Chart review, observational.

## Results

Thrombotic events have been associated with antiphospholipid antibodies in autoimmune diseases, including systemic lupus erythematosus (SLE). However, pulmonary thromboembolism (PTE) from deep vein thromboses (DVT) or in situ pulmonary arterial thrombosis is uncommon in rheumatic diseases, especially in children. The diagnosis and treatment of PTE may be delayed due to a paucity of symptoms or to symptoms attributed to more common manifestations such as pleuritis or pneumonia. We report findings in 6 children with PTE secondary to SLE (4), Systemic Sclerosis (SSc) (1) and Polyarteritis Nodosa (PAN) (1).

**Table 1:** 

Pt/Gender	1/F	2/F	3/M	4/F	5/M	6/M
Dx	SLE	SLE	SLE	SLE	SSc	PAN
	thrombocytopenia	nephritis (IV)	nephritis (V)	nephritis (IV)	PAH	CVA
Age at Dx (yr)	12.6	14.1	9.0	12.0	12.8	0.3
Age at PTE	15.2	14.8	16.8	12.6	16	6/4
Symptoms	leg pain	chest pain	chest pain	chest pain	chest pain	leg pain
		dyspnea	dyspnea	dyspnea	dyspnea	
DVT	+	-	-	-	-	+
Lupus AC	+	-	-	-	-	+/-
Anticardio AB	-	-	-	-	-	+
D-dimer (ng/ml)	647 (nl <220)	8770	1600	>10,000	n/a	>10,000
Albumin (g/dl)	4.7 (nl 3.7-5.5)	2.0	0.7	1.7	4.1	3.6
AT III (%)	105 (nl 77-132)	278	7/2	154	n/a	114
Fibrinogen (ng/dl)	718 (nl 180-394)	234	n/a	298	n/a	421

[PAH pulmonary arterial hypertension, CVA: cerebral vascular accident, AT III: anti-thrombin III, N/A: not available] All patients were treated with heparin and improved. No patient had any other genetic risk factors predisposing to thrombophilia.

## Conclusion

Although antiphospholipid antibodies are common in SLE, pulmonary arterial thrombosis is rare. These 4 cases of SLE represent only 1.7% of 234 pediatric lupus patients seen at Emory University over 18 years. Pulmonary thromboemboli may mimic pleuritis with effusion or pneumonia. Besides antiphospholipid antibodies, which were present in only 2 of these patients, other associated findings include nephrotic syndrome, elevated D-dimers and elevated fibrinogen levels. Recognition of PTE in pediatric patients with rheumatic diseases and prompt anti-coagulation therapy is important and potentially life-saving.

## Disclosure

Larry B. Vogler: None; Sheila Angeles-Han: None; Sampath Prahalad: None; Egla C. Rabinovich: None.

